# Off-label use of rituximab in patients with systemic lupus erythematosus with extrarenal disease activity: a retrospective study and literature review

**DOI:** 10.3389/fmed.2023.1159794

**Published:** 2023-05-25

**Authors:** Carla Sans-Pola, Immaculada Danés, Josep Àngel Bosch, Patricia Marrero-Álvarez, Josefina Cortés, Antònia Agustí

**Affiliations:** ^1^Department of Clinical Pharmacology, Vall d’Hebron Hospital Universitari, Vall d'Hebron Barcelona Hospital Campus, Barcelona, Spain; ^2^Department of Pharmacology, Therapeutics and Toxicology, Universitat Autònoma de Barcelona, Bellaterra, Spain; ^3^Clinical Pharmacology Research Group, Vall d’Hebron Institut de Recerca (VHIR), Vall d’Hebron Hospital Universitari, Barcelona, Spain; ^4^Department of Internal Medicine, Universitat Autònoma de Barcelona, Bellaterra, Spain; ^5^Pharmacy Department, Vall d’Hebron Hospital Universitari, Vall d'Hebron Barcelona Hospital Campus, Barcelona, Spain; ^6^Department of Internal Medicine, Vall d’Hebron Hospital Universitari, Barcelona Hospital Campus, Barcelona, Spain

**Keywords:** rituximab, off-label, systemic erythematosus lupus, CD20, effectiveness

## Abstract

**Introduction:**

Off-label rituximab is commonly used for patients with systemic lupus erythematosus (SLE) with extrarenal disease activity.

**Methods:**

The outcomes and tolerability of rituximab in adult patients with non-renal SLE treated at our hospital from 2013 to 2020 were described. Patients were followed-up until December 2021. Data were retrieved from electronic medical records. Response was classified into complete, partial or no response according to the Systemic Lupus Erythematosus Disease Activity Index 2000 (SLEDAI 2 K)-based definitions.

**Results:**

A total of 44 cycles were administered to 33 patients. Median age was 45 years and 97% were female. Median follow-up was 5.9 years (IQR 3.7–7.2). The most frequent symptoms that motivated rituximab use were thrombocytopenia (30.3%), arthritis (30.3%), neurological manifestations (24.2%) and cutaneous lupus (15.2%). After most treatment cycles a partial remission was achieved. The median SLEDAI-2 K score declined from 9 (IQR 5–13) to 1.5 (IQR 0–4) (*p* < 0.00001). The median number of flares significantly declined after receiving rituximab. Platelet counts significantly improved in patients with thrombocytopenia and patients with skin disorders or neurological manifestations also had a partial or complete response. Only 50% of patients with a predominant joint involvement had either a complete or a partial response. The median time to relapse after the first cycle was 1.6 years (95% CI, 0.6–3.1). Anti-dsDNA levels decreased significantly after rituximab from a median of 64.3 (IQR 12–373.9) to 32.7 (IQR 10–173), *p* = 0.00338. The most frequent adverse events were infusion-related reactions (18.2%) and infections (57.6%). All patients needed further treatment to maintain remission or to treat new flares.

**Conclusion:**

A partial or complete response was documented after most rituximab cycles in patients with non-renal SLE. Patients with thrombocytopenia, neurolupus, and cutaneous lupus had better response than those with a predominant joint involvement.

## 1. Introduction

Rituximab is a chimeric mouse/human monoclonal antibody that binds specifically to the transmembrane antigen CD20 located on B lymphocytes. It was initially approved by the European Medicines Agency (EMA) in 1998 for the treatment of patients with chemoresistant stage III-IV lymphoma. Since then, its indications have broadened and it is currently licensed for the treatment of non-Hodgkin’s lymphoma, chronic lymphocytic leukemia, rheumatoid arthritis, granulomatosis with polyangiitis and microscopic polyangiitis and pemphigus vulgaris (1). However, it is also often prescribed off-label for the treatment of other indications, such as patients with resistant systemic lupus erythematosus (SLE).

In 2009, the Spanish legislation regulated and classified drug use in special situations, including the use of medicines in unapproved conditions, the use of unmarketed drugs and compassionate use (2). Taking into account that off-label use may increase the hospital spending on drugs and overall risks, the Catalan Health Service released an Instruction in 2010 to regulate its use in Catalonia (3). According to this regulation, drug and therapeutics committees of each hospital are in charge of the evaluation of cases of drug use in special situations and need to be individually authorized by the medical director of each center.

A retrospective study published in 2013 described all the off-label rituximab requests received in the Vall d’Hebron University Hospital and observed a high number of requests for systemic connective tissue disorders (4). A subsequent prospective study of patients treated with off-label drugs in five public hospitals in Catalonia showed that the most frequently requested drug was rituximab, which was used in 22 different indications, including SLE (5).

SLE is an autoimmune disease that can cause a heterogeneous pattern of organ damage with different clinical characteristics, variable course, and prognosis. The optimal treatment for SLE remains uncertain (6). Current therapies include the use of antimalarial agents, glucocorticoids, and other immunosuppressive therapies, including some biologics. Hydroxychloroquine is recommended for all patients, unless contraindicated, and glucocorticoids can be used at doses and route of administration that depend on the type and severity of the organ involvement. In patients not responding to hydroxychloroquine, alone or in combination with glucocorticoids, or in patients unable to reduce glucocorticoid use at doses acceptable for chronic use, the addition of immunomodulatory or immunosuppressive agents such as methotrexate, azathioprine or mycophenolate should be considered. Also, in practice, immunosuppressive drugs are often used to avoid use of glucocorticoids and to achieve a better control of the disease. Cyclophosphamide is usually reserved for those patients with organ or life-threatening disease or as a rescue therapy in patients not responding to other immunosuppressive agents.

Some patients, however, have inadequate responses to standard-of-care and can be defined as patients with residual disease activity, glucocorticoid resistance and/or frequent relapses. The treatment options for these patients include the use of biologics. It is known that B-cells have a critical role in the pathogenesis of SLE and there is evidence to support beneficial effects of B-cell targeting agents (7–11). Belimumab, a monoclonal antibody that inhibits B-cell activating factor (BAFF), has shown positive results in randomized clinical trials (11, 12) and in real life setting studies (13). It is currently recommended for extrarenal disease with inadequate control to first-line treatments and has recently been approved for patients with lupus nephritis (6).

The results of the EXPLORER randomized controlled trial failed to show superiority of rituximab compared with placebo in patients with non-renal SLE (8). Some studies have shown efficacy in patients with severe autoimmune thrombocytopenia and haemolytic anemia (14–16). Also, some smaller open-labeled studies have reported a good response after rituximab (17–23). However, rituximab is currently only used off-label in patients with severe SLE refractory to other immunosuppressive agents, or in patients with contraindications to these agents.

The aim of this study is to assess the rate of response and tolerability of off-label use of rituximab in patients with resistant extrarenal SLE, as well as the clinical evolution of treated patients. Additionally, a thorough literature review of previously published observational studies regarding patients with SLE treated with rituximab was performed.

## 2. Materials and methods

A retrospective observational study of adult patients with extrarenal SLE treated with off-label rituximab at the Vall d’Hebron University Hospital from January 2013 to December 2020 was performed. The patients who received rituximab for the treatment of acute lupus nephritis were excluded from this study. Patients were identified from a register of the off-label drug requests received at the Pharmacy department. Patients were followed-up until December 2021. The study was conducted at the Clinical Pharmacology department, in collaboration with the Internal Medicine department.

A review of electronic medical records was carried out to obtain demographic data, clinical data, information on the clinical manifestation that motivated for rituximab use (clinical, biological, pathological and image data), dosage and treatment regimen of rituximab, previous and concomitant treatments, short-term and long-term rituximab treatment outcomes, and adverse events. This information was verified by consulting the clinicians responsible for the patient’s care. Study data were collected and managed using REDCap electronic data capture tools hosted at Vall d’Hebron Institut de Recerca (VHIR) (24, 25).

Treatment response was classified as complete remission, partial remission, or no response according to the current guidelines and Systemic Lupus Erythematosus Disease Activity Index (SLEDAI)-based definitions (26). The Definitions Of Remission In SLE (DORIS) Task Force recommends a single definition of remission in SLE based on SLEDAI (26). Complete remission was defined according to the 2021 DORIS definition (26). Partial remission was defined as a 50% improvement in Systemic Lupus Erythematosus Disease Activity Index 2000 (SLEDAI-2 K) compared to baseline. When none of these criteria were met, the outcome was classified as no response.

Additionally, among patients with autoimmune thrombocytopenic purpura, a complete response was defined as a platelet count of ≥100,000 platelets/mm^3^ and a partial response as 20,000 - < 100,000 platelets/mm^3^. No change in the platelet count or a platelet count of <20,000 platelets/mm^3^ was considered as no response. An improvement of the hemoglobin and/or white cell counts compared to baseline was defined as a partial response and a normalization was defined as a complete response. No change in hemoglobin and/or white cell counts was considered as no response. Responses in other clinical manifestations, such as cutaneous, articular, and neurological manifestations, were defined as an improvement (partial response) or disappearance (complete response) compared to baseline as reported in the electronic medical records. No clinical change was considered as no response.

Treatment outcomes were assessed 2 to 6 months after each rituximab treatment cycle. Serological markers were assessed at baseline and after rituximab treatment including complement (C3 and C4; normal values 85–180 and 10–40 mg/dl, respectively), erythrocyte sedimentation rate (ESR; normal <20 mm/h), double-stranded DNA antibodies (Anti-dsDNA; negative <27 UI/mL, indeterminate 27–35 UI/mL, positive >35 UI/mL), and immunoglobulin G (IgG; normal 700–1,600 mg/dl). Circulating CD19+ B-cell levels were also obtained when available.

Disease flares were defined according to the Safety of Estrogens in Lupus Erythematosus National Assessment (SELENA)-SLEDAI flare index considering changes in SLEDAI score and/or individual manifestations, changes in treatment, need for hospitalization and/or changes in PhGA (27).

All patients treated with rituximab in the Vall d’Hebron University Hospital receive premedication before rituximab infusions, which consists of paracetamol, methylprednisolone, and antihistaminic drugs, and after, they all receive prophylactic treatment with trimethoprim/sulfamethoxazole to prevent *Pneumocystis jirovecii* infection.

Adverse events were classified according to the Medical Dictionary for Regulatory Activities MedDRA (28) and were assessed according to the algorithm of the Spanish Pharmacovigilance System (29, 30). The International Classification of Diseases 11th revision (ICD-11) was used to classify medical indications for rituximab use (31).

This study was conducted according to international ethical recommendations and was approved by the local Research Ethics Committee following the national directives related to observational studies. Patient consent was waived because the study was retrospective, containing deidentified data.

Statistical analysis of categorical and continuous variables was performed by proportions, median and interquartile range (IQR), respectively. Statistical differences were assessed using the Wilcoxon signed-rank test. Significance was set at a level of 0.05 and was two-tailed. Kaplan–Meier curves were used to estimate the time to flare. The analysis was performed using R software 4.1.3 (32).

A search in PubMed was performed using the MeSH terms “Rituximab” and “Lupus Erythematosus, Systemic” together with free text “rituximab” and “Systemic Lupus Erythematosus,” up to December 2022. Previously published observational studies in English and Spanish were included. Clinical trials, case series, case reports and book chapters were excluded.

## 3. Results

During the study period, 44 requests for off-label use of rituximab were received for 33 patients presenting non-renal manifestations of SLE. All requests were approved and administered.

The median age of patients was 45 years (IQR 36–55) and 32 (97%) were female. Their baseline characteristics can be seen in Table 1.

**Table 1:** Baseline characteristics.

Baseline characteristics	Patients with extrarenal SLE disease activity (*n* = 33)
Age (years), *median (IQR)*	45 (36–55)
Sex	
Women, *n (%)*	32 (97.0%)
Men, *n (%)*	1 (3.0%)
Tobacco smoking, *n (%)*	5 (15.2%)
Alcohol consumption, *n (%)*	1 (3.0%)
Comorbidities	
Hypertension, *n (%)*	6 (18.2%)
Antiphospholipid syndrome, *n (%)*	5 (15.2%)
Dyslipidemia, *n (%)*	2 (6.1%)
Type 2 diabetes, *n (%)*	1 (3.0%)
Main clinical manifestations*	
Thrombocytopenia, *n (%)*	10 (30.3%)
Joint involvement, *n (%)*	10 (30.3%)
Neurological manifestations, *n (%)*	8 (24.2%)
Skin manifestations, *n (%)*	5 (15.2%)
Neutropenia, *n (%)*	1 (3.0%)
Hemolytic anemia, *n (%)*	1 (3.0%)
Optic neuritis, *n (%)*	1 (3.0%)
Number of organs involved, *median (IQR)*	2 (1–2)
1 organ involved, *n (%)*	10 (30.3%)
2 organs involved, *n (%)*	15 (45.5%)
>2 organs involved, *n (%)*	8 (24.2%)
Serological markers	
C3 (mg/dL), *median (IQR)*	84.5 (65.7–109)
C4 (mg/dL), *median (IQR)*	12.8 (7–20)
ESR (mm/h), *median (IQR)*	28 (18–39)
Anti-dsDNA (IU/mL), *median (IQR)*	54.8 (12–323)
IgG (g/L), *median (IQR)*	1,255 (1,020 -1,661)
SLEDAI-2 K score, *median (IQR)*	9 (5–13)
SLE flares before RTX, *median (IQR)*	4 (3–5)
Disease duration (years), *median (IQR)*	7.1 (2.6–11.8)
Previous treatment with antimalarial agents, *n (%)*	18 (54.5%)
N° of previous immunosuppressive agents, *median (IQR)*	5 (3–6)
Previous immunosuppressive agents	
Oral corticosteroids*, n (%)*	30 (90.9%)
IV corticosteroids, *n (%)*	10 (30.0%)
Mycophenolate mofetil, *n (%)*	26 (78.8%)
Tacrolimus, *n (%)*	18 (54.5%)
Azathioprine, *n (%)*	10 (30.0%)
Ciclosporin, *n (%)*	7 (21.2%)
Etanercept, *n (%)*	6 (18.2%)
Cyclophosphamide, n (%)	5 (15.2%)
Belimumab, *n (%)*	1 (3.0%)
Dose of oral corticosteroids (mg), *median (IQR)*	5 (5–13.8)
Previous treatment with IVIG, *n (%)*	8 (24.2%)

Anti-dsDNA, anti-double stranded DNA antibodies; C3, complement component 3; C4, complement component 4; ESR, erythrocyte sedimentation rate; IgG, Immunoglobulin G; IQR, interquartile range; IV, intravenous; IVIG, intravenous immunoglobulin; RTX, rituximab; SLE, Systemic Lupus Erythematosus; SLEDAI-2 K, Systemic Lupus Erythematosus Disease Activity Index 2000. *When rituximab was requested, patients could have more than one clinical manifestation.

When rituximab was requested, patients could have more than one clinical manifestation. The most frequent symptoms and/or signs that motivated rituximab use were thrombocytopenia (*n* = 10; 30.3%. Six patients had symptomatic thrombocytopenia. The most common symptoms were petechiae and hematomas), arthritis (*n* = 10; 30.3%) and neurological manifestations (*n* = 8; 24.2%). Other indications were cutaneous lupus (*n* = 5; 15.2%), neutropenia (*n* = 1; 3%), hemolytic anemia (*n* = 1; 3%) and optic neuritis (*n* = 1; 3%). There were 5 patients (15.2%) with concomitant antiphospholipid syndrome, and 2 (6.1%) with autoimmune hepatitis. Five patients (15.2%) also had renal involvement at some point, but they did not have renal activity when rituximab was requested and administered. The most frequent comorbidity was hypertension. Median follow-up was 5.9 years (IQR 3.7–7.2).

Most patients had received previous immunosuppressive therapies with a median of 5 (IQR 3–6) different agents and had refractory or relapsing disease. The median time from diagnosis to the first rituximab cycle was 7.1 years (IQR 2.6–11.8). The median number of rituximab cycles for each patient was 1 (IQR 1–2). Twenty-one patients (63.6%) had only one cycle of rituximab, and 12 (36.4%) received more than one cycle. The median number of years between the first course and the second course was of 2.5 years (IQR 1.6–3.6). The main organic manifestations of the patients that received more than one course of rituximab (n = 12) were neurologic manifestations (5; 41.7%), joint involvement (4; 33.3%), and thrombocytopenia (3; 25.0%).

Thirty-five cycles (79.5%) consisted of the administration of two doses of 1,000 mg given intravenously with a 2-week interval, seven (15.9%) were low-dose rituximab regimens consisting of four weekly doses of 100 mg given intravenously (total dose 400 mg), and two (4.5%) cycles were adjusted to body surface (375 mg/m^2^ for 4 weeks). All patients who received low-dose rituximab cycles had thrombocytopenia as their main disease manifestation.

### 3.1. Disease activity

Response was assessed after a median of 3 months (IQR 2–4). After the majority of the 44 rituximab cycles a partial remission was achieved (n = 31; 70.5%); however, no response was observed in 11 (25.0%) of them. A complete remission was achieved in two (4.5%). The median SLEDAI-2 K score declined from 9 (IQR 5–13) at baseline to 1.5 (0–4) after rituximab treatment (*p* < 0.00001).

The median number of flares before (from diagnosis to the first cycle) and after receiving treatment with rituximab was 4 (IQR 3–5) and 2 (IQR 0–3), respectively (*p* = 0.00008), with a rate of 48.4 flares per 100 patient-years and 37.3 flares per 100 patient-years, respectively. Eleven patients (33.3%) had flares in the main clinical manifestation that led to the use of rituximab.

Most patients (66.7%) had a partial remission after rituximab treatment, however 7 (21.2%) never responded, Table 2. Among the 12 patients that received more than one course of rituximab, one patient responded completely after the first course, and partially after the second. Nine patients had a partial response after the first course and the majority (7/9) also responded partially after the second course (the remaining two patients had no response). Two patients had no response after the first course, but still received one more rituximab cycle to which they did equally not respond.

**Table 2:** Observed treatment outcomes.

Patients, *n*	33
Always CR, *n (%)*	1 (3.0%)
Always PR, *n (%)*	22 (66.7%)
Always NR, *n (%)*	7 (21.2%)
Some response after the first cycle but NR after subsequent cycles, *n (%)*	2 (6.1%)
CR after first cycle, PR after subsequent cycles, *n (%)*	1 (3.0%)

CR, complete remission; NR, no response; PR, partial remission.

Among patients with thrombocytopenia, nine (90%) had a complete or partial response after rituximab. Seven patients with thrombocytopenia (70%) received low-dose rituximab (100 mg weekly for 4 weeks), from these, a complete response was observed in one and partial response in five. The median platelet count before and after receiving treatment with rituximab was 48,000 (IQR 14,000-60,000) and 188,000 (IQR 119–213), respectively (*p* = 0.00148). Four patients had baseline platelet counts lower than 20,000 platelets/mm^3^. Some patients only had thrombocytopenia as a clinical manifestation; thus, the change in their SLEDAI-2 K score was minimal or even inexistent. However, among the total of patients with thrombocytopenia there was a decrease in the median SLEDAI-2 K score from 3.5 (IQR 1.5–5) to 1 (IQR 0–3) because some of them had other manifestations. Table 3 shows change in disease activity before and after treatment with rituximab.

**Table 3:** Before and after disease activity, treatment, and serological markers.

	Thrombocytopenia *n* = 10		Joint involvement *n* = 10		Cutaneous lupus *n* = 5		Neurolupus *n* = 8	
	Before	After	Before	After	Before	After	Before	After
SLEDAI-2 K score, *median (IQR)*	3.5 (1.5–5)	1 (0–3)	12 (7.3–15.8)	4 (2.5–6)	12 (12–13)	5 (2–8)	11 (8–16)	0 (0–2)
SLE flares, *median (IQR)*	4 (3.3–5)	1.5 (0.3–3.8)	5 (4–6.5)	3 (2–4.8)	5 (5–5)	2 (2–5)	3 (2–5.5)	2 (1–2.5)
Treatment with antimalarial agents, *n (%)*	6 (60%)	5 (50%)	6 (60%)	7 (70%)	2 (40%)	2 (40%)	2 (25%)	3 (37.5%)
Number of IS agents, *median (IQR)*	3.5 (3–5.8)	3 (2.3–3.8)	5 (4–6)	4 (3–5)	4 (4–5)	3 (2–3)	3 (2–5)	2 (2–3.5)
IVIG, *n (%)*	5 (50%)	2 (20%)	2 (20%)	0	0	1 (20%)	1 (12.5%)	1 (12.5%)
Oral corticosteroids*, n (%)*	10 (100%)	7 (70%)	10 (100%)	8 (80%)	5 (100%)	4 (80%)	7 (87.5%)	5 (62.5%)
IV corticosteroids, *n (%)*	2 (20%)	2 (20%)	1 (10%)	5 (50%)	2 (40%)	1 (20%)	4 (50%)	1 (12.5%)
C3 (mg/dL), *median (IQR)*	108 (79.3–136)	114 (97.9–129)	69.70 (59.9–99.8)	83 (70.6–110.5)	89.9 (84–102)	103 (90.2–119)	65.7 (51–89.6)	84 (74.5–98.5)
C4 (mg/dL), *median (IQR)*	15.65 (9.9–19.6)	19.3 (16.9–23.7)	9.14 (7.31–18.5)	15.4 (12–19.7)	12.8 (10–16)	16 (15.9–23.5)	13.9 (6.5–15.8)	17.3 (12.9–21.4)
ESR (mm/h), *median (IQR)*	29 (18.3–37)	15 (14–20)	31.5 (22–57.3)	20 (17–24.5)	20 (20–42)	20 (18–24)	20 (18.5–33)	24 (19.5–34.5)
Anti-dsDNA (IU/ml), *median (IQR)*	47.53 (12.7–141.5)	12 (10–70.8)	294.28 (37.71–459.3)	125 (36.5–347.8)	358 (50.2–591.9)	125 (50.5–146.2)	99 (32.4–420.4)	28.6 (10.5–167.2)
IgG (g/L), *median (IQR)*	1,260.5 (1,128.5-1,466.5)	1,287 (1,040-1,546)	1,788.50 (1,688.5-1,951.3)	1,673 (1,344.5-2,008)	1,163 (784–1,365)	1,009 (607–1,402)	1,025 (956–1,239)	1,041 (918.5–1,349.5)

Anti-dsDNA, anti-double stranded DNA antibodies; C3, complement component 3; C4, complement component 4; ESR, erythrocyte sedimentation rate; IgG, Immunoglobulin G; IQR, interquartile range; IS, immunosuppressive; IVIG, intravenous immunoglobulin; IV, intravenous; RTX, rituximab; SLE, systemic lupus erythematosus; SLEDAI-2 K, Systemic Lupus Erythematosus Disease Activity Index 2000.

Four in 5 patients (80%) with skin disorders and all patients (100%) with neurological manifestations had either a complete or a partial response after rituximab cycles. However, only 50% (5 in 10) of patients with a predominant joint involvement had some response to rituximab.

All patients but one received other concomitant treatments to treat SLE flares. The median number of concomitant therapies was 3 (IQR 2–3), and the most frequent were high-dose intravenous and/or oral glucocorticoids (42.4 and 72.7%, respectively).

All patients needed further immunosuppressive therapies during follow-up to maintain remission or to treat new flares. The median number of immunosuppressive agents after rituximab treatment was 3 (IQR 2–5). Nineteen patients (57.6%) started treatment with new immunosuppressive agents after rituximab. Table 4 shows concomitant therapies and treatments after rituximab.

**Table 4:** Concomitant treatment and therapies after rituximab.

	Patients with extrarenal SLE disease activity treated with rituximab (*n* = 33)
Concomitant treatment	
N° of concomitant immunosuppressive agents, *median (IQR)*	3 (2–3)
Concomitant immunosuppressive agents, *n (%)*	
Prednisone	24 (72.7%)
Mycophenolate mofetil	16 (48.5%)
High-dose methylprednisolone	14 (42.4%)
Tacrolimus	10 (30.3%)
Dose of oral corticosteroids (mg), *median (IQR)*	10 (5–20)
IVIG, *n (%)*	2 (6.1%)
Antimalarial agents, *n (%)*	9 (27.3%)
Treatment after RTX	
N° of IS agents after RTX, *median (IQR)*	3 (2–5)
IS agents, *n (%)*	
Prednisone	27 (81.8%)
Mycophenolate mofetil	25 (75.8%)
Tacrolimus	12 (36.4%)
Cyclophosphamide	10 (30.3%)
High-dose methylprednisolone	6 (18.2%)
Ciclosporin	4 (12.1%)
Belimumab	2 (6.1%)
Dose of oral corticosteroids (mg), *median (IQR)*	5 (2.5–5)
New IS agents, *n (%)*	19 (57.6%)
IVIG, *n (%)*	3 (9.1%)
Antimalarial agents, *n (%)*	13 (39.4%)

IQR, interquartile range; IS, immunosuppressive; IVIG, intravenous immunoglobulin; RTX, rituximab; SLE, Systemic Lupus Erythematosus.

**Figure 1:**
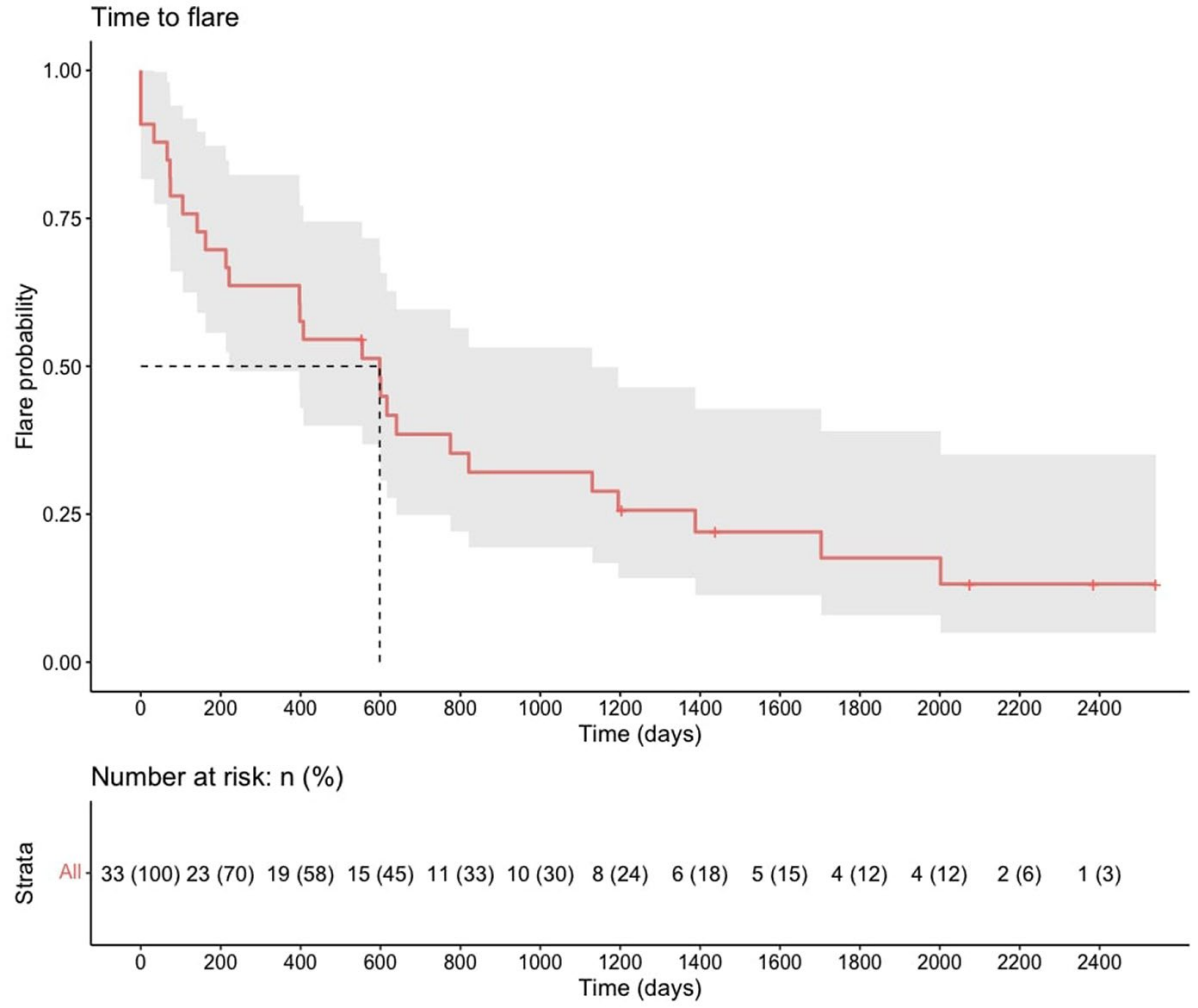
Time-to-flare after the first course of rituximab.

The median time to relapse after the first cycle of rituximab was 598 days (95% CI, 221–1,130) [1.6 years (95% CI, 0.6–3.1); Figure 1].

### 3.2. Serological markers of disease activity

#### 3.2.1. Inflammatory markers

At baseline, 19 (57.6%) and 3 (9.1%) patients had positive and intermediate anti-dsDNA levels assessed by enzyme linked immunosorbent assay (ELISA), respectively. As seen in Table 5, anti-dsDNA levels decreased significantly after treatment with rituximab. Seventeen (51.5%) and 13 (39.4%) patients had decreased C3 and C4 levels at baseline, respectively. Thirteen (39.4%) patients had decreased levels of both C3 and C4. Although complement levels seem to increase after rituximab, the difference did not reach statistical significance. Before and after levels for other markers can be seen in Tables 4, 5 for all patients and according to their main SLE manifestation, respectively.

**Table 5:** Serological markers of disease activity before and after treatment with rituximab for all patients.

Serological markers	Before RTX, *median (IQR)*	After RTX, *median (IQR)*	*p*
IgG (g/L)	1,259.5 (1,005–1,743.5)	1,236 (944.8–1,533.8)	0.005
Anti-dsDNA (IU/mL)	64.3 (12–373.9)	32.7 (10–173)	0.003
ESR (mm/h)	28 (16.5–43)	20 (14–29)	0.009
C3 (mg/dL)	83.3 (62.9–104.8)	95.5 (76.4–117.3)	0.459
C4 (mg/dL)	12.4 (7–18.9)	17.2 (11.1–23.2)	0.091

Anti-dsDNA, anti-double stranded DNA antibodies; C3, complement component 3; C4, complement component 4; ESR, erythrocyte sedimentation rate; IgG, Immunoglobulin G; IQR, interquartile range; RTX, rituximab.

#### 3.2.2. Circulating CD19-positive B lymphocytes

Circulating CD19-positive B cells were not routinely identified at baseline for these patients; thus, no comparison can be made after receiving treatment with rituximab. The median CD19-positive peripheral B cell proportion at 2–4 months after rituximab cycles was 0.05% (IQR 0–0.14), and the median count was 0.0 × 10^9^/L (IQR 0.0–0.005).

### 3.3. Adverse events

Nine patients (27.6%) had adverse events that were probably related to rituximab during the study period (Table 6). One patient had two adverse events. Six patients (18.2%) had infusion-related adverse reactions, such as skin rash, uvular edema, and dizziness during the infusion. One patient had an anaphylactic shock during the infusion that required medical attention and treatment with adrenaline and intravenous dexchlorpheniramine. No further rituximab cycles were administered to this patient. There was also one serum sickness-like reaction, and one patient with persistent hypogammaglobulinemia.

**Table 6:** Summary of adverse events.

Adverse event	*n* (%)
Infusion-related reaction	6 (18.2%)
Fever	1 (3.0%)
Hypogammaglobulinemia	1 (3.0%)
Serum sickness-like reaction	1 (3.0%)
Anaphylactic shock	1 (3.0%)
Infections	
Respiratory tract infection	9 (27.3%)
Urinary tract infection	8 (24.2%)
Gastrointestinal infection	2 (6.1%)
COVID-19	1 (3.0%)
Sepsis with septic shock	1 (3.0%)

Furthermore, 19 patients (57.6%) had one or more infectious complications during follow-up, mostly respiratory and urinary tract infections, and seven (21.2%) required admission to hospital. Nine patients (27.3%) had reported infections before rituximab, being the most common urinary tract infections, and three of them required admission to hospital.

## 4. Discussion

The results of this study show that off-label rituximab among our patient cohort was mainly used for the treatment of thrombocytopenia, arthritis, neurological manifestations, and cutaneous lupus. Some response was achieved after most rituximab cycles and there was a significant decline in the SLEDAI-2 K score after rituximab compared to baseline scores. Most patients had received immunosuppressive treatments before the first rituximab request and had refractory or relapsing disease. There was a significant decrease in the number of disease flares after rituximab use. Patients with thrombocytopenia had a significant improvement of platelet counts after receiving rituximab albeit some did not have an important difference in SLEDAI-2 K scores before and after treatment. This is because thrombocytopenia does not have a big impact in the SLEDAI-2 K score. Patients with skin disorders or neurological manifestations also had a partial or complete response. However, only half of patients with a predominant joint involvement had either a complete or a partial response. All patients required further immunosuppressive treatment after rituximab either to maintain remission or to treat new flares.

Some previous studies have been conducted on patients with SLE and idiopathic thrombocytopenic purpura (SLE-ITP) treated with rituximab and have been consistent with their results (33–36). Currently, rituximab is recommended in the guidelines for patients who are refractory to corticosteroids and other immunosuppressants (27). A recent study described the use of low-dose rituximab for severe refractory SLE-ITP with a response rate of 60% (37). However, a large proportion of patients failed to respond to rituximab, with no improvement after dose and interval adjustment. The cause for the failure was unclear. Analysis of spleen samples from patients who failed rituximab showed that rituximab completely depleted peripheral B cells and had no effect on splenic plasma cells, while the remaining plasma cells continued to secrete anti-platelet antibodies.

The median time from the first administration of rituximab to relapse was of almost 20 months in our study. Other similar observational studies have reported variable results (15, 21, 38–40). The study by Vital et al. showed a wide variability and suggested that time-to-relapse could be divided into two phases: 14 patients relapsed within the subsequent 12 months after rituximab and were classified as “early relapse,” and the remaining (*n* = 25) had a much longer time-to-relapse (median of 33 months) and were classified as “late relapse” (41).

Our results show that there was a significant decrease in anti-dsDNA antibody levels after treatment with rituximab, compared with baseline levels, which is consistent with the known biologic effect of rituximab treatment already observed in previous studies. However, there was no significant difference in C3 and C4 levels or other serological markers of disease activity. Previous evidence has showed that the association between these markers and response to treatment and later events such as the risk of relapse and subsequent morbidity and mortality is not clear. Thus, the DORIS 2021 Task Force did not recommend the inclusion of serology (anti-dsDNA and complement) in the definition of remission (26).

The interpretation of these results should be carried out considering that most patients were refractory to or dependent on other treatments, and that they were receiving concomitant immunosuppressive agents, which means that they had an active disease with moderate to severe symptoms.

Available evidence for using rituximab to treat these patients is variable. The EXPLORER trial, published in 2010, assessed the efficacy and safety of rituximab versus placebo in 257 patients with moderately-to-severely active extrarenal SLE over 52 weeks (8). No significant differences were observed between rituximab and placebo in the primary and secondary efficacy endpoints; however, a beneficial effect of rituximab was noted in the African American and Hispanic patients. Some explanations have been proposed to justify the trial failure, such as the association of immunosuppressive medication and even patient heterogeneity and inappropriate design of endpoints (42, 43). Furthermore, it is worth noting that patients who had organ-threatening lupus requiring significant use of glucocorticoids or recent treatment with cyclophosphamide or a calcineurin inhibitor, and those who had received previous treatment with B cell-targeted drugs were excluded from the EXPLORER trial. As we have seen, this is far from the reality of patients with non-renal SLE treated with rituximab in clinical practice. The patients included in our study had a greater previous exposure to immunosuppressive agents. This suggests that the results from available clinical trials may not be applicable in patients with a more severe disease, such as the ones included in this study. Additionally, physicians often assess the clinical response to rituximab according to the improvement, disappearance, or no change in the main organ manifestations, as well as the change in disease activity indexes. Thus, the results from observational studies that also include this assessment can be more relevant to real world settings. It is suggested that rituximab, if used earlier, might offer significant advantages in some patients in the duration of active disease, and avoiding side effects of multiple immunosuppressive agents and chronic use of corticosteroids (44).

Unfortunately, due to the lack of indication, rituximab might not be available for similar patients with SLE from other settings that do not have an easy access to off-label prescription (45). Our experience with the management of these patients and off-label use of medicines, such as rituximab, can add to the existing evidence and might help with decision taking in other centers that can encounter patients with similar needs. Table 7 summarizes the main characteristics of previously published observational studies that include data from patients with SLE that were treated with rituximab. Their main results can be seen in Supplementary Table S1. Even after the publication of the results from the EXPLORER trial, some patients have still received rituximab in clinical practice and some results from observational studies have been published. The studies published by Terrier et al. and Fernández-Nebro et al. both show a significant reduction in the mean SELENA-SLEDAI scores, and both observed significant results in patients with thrombocytopenia, joint involvement, and cutaneous lupus (15, 21). Our results showed the best responses in patients with thrombocytopenia (90%), skin involvement (80%) or neurological manifestations (100%). However, only half of patients with joint involvement had some degree of response to rituximab. There are multiple reasons that can explain these differences, including the small number of patients, patient heterogeneity, variations in the assessment of outcomes and the use of different disease activity scores. Moreover, in our study the clinical and/or laboratory response was assessed for each main organ involvement, in addition to the SLEDAI score change. This was not done equally in previous observational studies; thus, some difference can be expected.

**Table 7:** Summary of previously published observational studies.

References	Study design	*n*	Assessment of response	Organ-specific results
Gottenberg et al. (46)	Retrospective cohort	13	SLEDAI	No
Smith et al. (38)	Prospective cohort	11	BILAG	No
Tokunaga et al. (47)	Prospective cohort	10	SLEDAI + clinical change in neuropsychiatric manifestations	Yes (NP)
Jónsdóttir et al. (48)	Prospective cohort	16	SLEDAI and BILAG	No
Lu et al. (18)	Prospective cohort	50	BILAG	No
Catapano et al. (39)	Prospective cohort	31	BILAG	No
Terrier et al. (15)	Prospective cohort	136	SELENA-SLEDAI + clinical change + platelet and hemoglobin level change	Yes (C, A, K, B, S, CNS, H, PN, L)
Vital et al. (41)	Prospective cohort	39	BILAG	No
Turner-Stokes et al. (49)	Prospective cohort	18	BILAG	No
Pinto et al. (50)	Prospective cohort	42	SELENA-SLEDAI + clinical change + platelet and hemoglobin level change	Yes (K, NP, B, MS, H, L)
Fernández-Nebro et al. (21)	Retrospective cohort	128	SELENA-SLEDAI. No additional organ-specific assessment of clinical change.	Yes (MS, C, K, B, N, H, L, other)
Witt et al. (51)	Retrospective cohort	85	SELENA-SLEDAI + presence of disease manifestations	Yes (A, B, C, CNS, K, MS, N, S)
Gómez et al. (52)	Retrospective cohort	20	SLEDAI	No
Cassia et al. (40)	Retrospective cohort	147	ECLAM + physician assessment.	No

*A, articular; B, blood; C, cutaneous; CNS, Central nervous system; H, heart; K, kidney; L, lung; MS, musculoskeletal; N, neurological; NP, neuropsychiatric; PN, peripheral neuropathy; S, serositis. BILAG, British Isles Lupus Assessment Group; ECLAM, European Consensus Lupus Activity Measurement; SELENA-SLEDAI, Safety of Estrogens in Lupus Erythematosus National Assessment- Systemic Lupus Erythematosus Disease Activity Index; SLEDAI:, Systemic Lupus Erythematosus Disease Activity Index.

Since circulating CD19+ B cells were not routinely identified at baseline for all patients and were not assessed in every single follow-up visit after the first one in clinical practice, it is difficult to see their evolution and change over time and their association with disease remission and relapse. CD19+ B cell depletion is a marker for rituximab’s biologic effect and, despite some authors (41) have found an association with disease remission and clinical response, this has not always been the case. The effect of rituximab on B cells is transient and some variability across patients in the response is expected, which can be partially explained by the reduction of the frequency of specific B cell subsets and phenotypes that can differ between patients (53). The study by Jónsdóttir et al. showed that patients with low baseline levels of CD19-positive lymphocytes can also respond to depletion with rituximab, due to the lack of correlation between peripheral blood and the rest of the population of CD19-positive B cells in the lymphoid tissues (20). Another study did not observe any relationship between the duration of peripheral B cell depletion and disease flare (54). Other studies have found that B cell depletion leads to an increase in B-cell activating factor (BAFF) levels in the spleen and serum, which promotes plasma cell survival and differentiation into long-lived plasma cells. These long-lived plasma cells may be the reason for the failure of rituximab treatment (55). In the EXPLORER trial, approximately 9.5% of the treated patients did not achieve B cell depletion on day 15, with maintenance of increased levels until day 84. An *ad hoc* analysis removing patients with incomplete B cell depletion did not change de primary outcome (8).

The adverse events identified in our study are similar to the already known safety profile for rituximab and to those previously published in clinical trials and observational studies (8, 17–23). More than half of the patients had one or more infectious complications during the study period. However, once more it is worth noting that most patients were receiving other immunosuppressive agents concomitantly. In the EXPLORER trial, the proportion of patients with infectious adverse events was similar between the two groups (rituximab 82.2%; placebo 83.0%). Infusion-related reactions are a known adverse event related to rituximab administration. In the EXPLORER trial, a 13.6% of patients had an infusion-related reaction (18.2% in our study). It is worth mentioning that secondary inefficacy related to infusion reactions and anti-drug antibodies occur in approximately 14% of SLE patients receiving repeated rituximab courses (41).

Some patients in our study were treated with low-dose (100 mg, 4 doses) rituximab. It has been suggested to reduce the immunosuppressive burden and, thus, the risk of adverse reactions and infections. However, the available data on its efficacy is still limited. Only few studies have evaluated the role of low-dose rituximab in SLE, and these were only in patients with lupus-induced thrombocytopenia (56, 57).

Other B-cell depletion agents that have data in non-renal SLE patients are obinutuzumab, epratuzumab, and ofatumumab (58–60). Some data support the use of B-cell survival factor inhibitors, such as belimumab, which was already approved by the EMA for SLE (10, 61). Other similar agents that have data in non-renal SLE are atacicept, bislimimod and tabalumab, but none has been approved for now (62–64). Additionally, anifrolumab, a human immonuglobulin G1 kappa monoclonal antibody that binds to the type 1 interferon receptor, has recently been approved by the EMA for patients with autoantibody-positive SLE (65). Other therapies that have shown some results include plasma cell inhibition (bortezomib and daratumumab) (66–68), tyrosin kinase inhibition (tofacitinib and fenebrutinib) (69, 70) and forigerimod, a CD4 T-cell modulator, which is currently being evaluated in phase III clinical trials (71, 72). Iberdomide has been evaluated in a phase II clinical trial with promising results compared to placebo (73). Combination therapy with rituximab plus belimumab has obtained encouraging results in recent clinical trials both for patients with lupus nephritis and for patients with non-renal SLE (74–76). The use of CD19 CAR-T cell treatment in five patients with SLE has shown promising results and clinical trials are ongoing (77, 78).

It is worth noting that although belimumab is currently recommended for patients with extrarenal disease (61); it was scarcely used in our center. Most patients did not comply with the criteria for use established by the Catalan Health Service to improve belimumab efficiency and effectiveness, which were: adult patients with SLE with a score of >10 in the SELENA-SLEDAI scale at the moment of prescription, low C3 levels (< 90 mg/dl) and C4 levels (< 10 mg/dl), positive anti-dsDNA levels, who were receiving immunosuppressive treatment (azathioprine, mycophenolate, cyclophosphamide, methotrexate, calcineurin inhibitors, leflunomide) and/or high-dose corticoids during the last 6 months without response, and without active renal disease or neurological involvement.

The main limitation of this study is that it is an observational study with a retrospective design and without a control group. This implies a risk of bias and some missing results in clinical records that required handling in some analysis. Only one center was included in our study; thus, the results cannot be extrapolated to other hospitals in other geographical areas. Additionally, since the criteria to evaluate the clinical response in the main clinical manifestations were based on the treating physician’s judgment, data on poor response might be increased.

However, the main strength of our study is that the participating center is a tertiary referral hospital with a high level of complexity and all patients are followed by experts in SLE and assessed and managed following the same criteria. Each patient was usually followed by the same expert in each visit, which reduces the variability in the assessment of the treatment outcomes. Furthermore, all rituximab requests were evaluated and approved by the hospitals’ drug and therapeutics committee, which guarantees an additional thorough assessment of each patient.

## 5. Conclusion

Thrombocytopenia, arthritis, and neurological manifestations were the main symptoms that motivated off-label rituximab use in patients with extrarenal SLE in our center during the study period. Three-quarters of the treated patients achieved a response, but almost all of them were partial. There were clinical improvements in platelet counts, skin disorders and neurological symptoms, and a significant reduction in disease flares. However, most patients needed further immunosuppressive treatment to maintain remission or to treat new flares. Although rituximab can be an option for some patients with refractory and severe disease, future data from other anti-CD20 agents or emerging therapies is needed to clarify the optimal treatment for patients with non-renal SLE.

## Data availability statement

The raw data supporting the conclusions of this article will be made available by the authors, without undue reservation.

## Ethics statement

The studies involving human participants were reviewed and approved by the Research Ethics Committee, Vall d’Hebron University Hospital. Written informed consent for participation was not required for this study in accordance with the national legislation and the institutional requirements.

## Author contributions

AA, ID, JB, and JC: conceptualization. AA, ID, and CS-P: methodology. CS-P: statistical analysis. CS-P, AA, ID, JB: investigation. CS-P: writing-original draft preparation. AA, ID, JB, PM-Á, JC, and CS-P: writing-review and editing. AA, ID, and JB: supervision. All authors have read and agreed to the published version of the manuscript.

## Funding

This research did not receive any specific grant from funding agencies in the public, commercial, or not-for-profit sectors.

## Conflict of interest

The authors declare that the research was conducted in the absence of any commercial or financial relationships that could be construed as a potential conflict of interest.

## Publisher’s note

All claims expressed in this article are solely those of the authors and do not necessarily represent those of their affiliated organizations, or those of the publisher, the editors and the reviewers. Any product that may be evaluated in this article, or claim that may be made by its manufacturer, is not guaranteed or endorsed by the publisher.
