# Supplementary material for: Off-label use of rituximab in patients with systemic lupus erythematosus with extrarenal disease activity: a retrospective study and literature review
Source: Front Med (Lausanne). 2023 May 25;10:1159794. doi: 10.3389/fmed.2023.1159794 (PMC10248418; doi:10.3389/fmed.2023.1159794)
Supplement: Supplementary file 1 [file Table_1.DOCX]

Supplementary Table S1. Previously published observational studies

| **Reference, study design, year** | **n, patient**  **characteristics*** | **Variables** | **Main results** |
| --- | --- | --- | --- |
| **Gottenberg et al. 2005**  **Retrospective cohort** | n = 13  Female: 10 (79.9%)  Myocarditis/Evans: n=1; Pericarditis: n=1; AIHA: n=1; CNS: n=1; Skin: n=2; Articular: n=3 ; Vasculitis: n=2; Autoimmune thrombocytopenia: n=2; Kidney: n=2 | Decrease of 50% (PR) or more of the initial DAS28 and SLEDAI values.  CR defined as SLEDAI value between 0 and 12.  PR defined as a decrease of ≥50% of the initial SLEDAI  Index: SLEDAI | **CR**: 7/13 (53.8%)  **CR and PR**: 9/13 (69.2%)  **Remission of patients without renal disease:** 7/9 (77.7%)  **Mean (±SD) SLEDAI:** 17±7 (range 3 - 28) to 5±6 (range 0 - 20) (p<0.002) |
| **Smith et al. 2006**  **Prospective cohort** | n = 11  Female: 10 (90.9%)  Median age: 43 years  Skin: n=11; Articular: n=11; Kidney: n=6; CNS: n=6; Lung: n=5; Ocular: n=2; Blood: n=1; Antiphospholipid syndrome: n=1; Gut: n=1; Heart: n=1 | CR required the absence of BILAG A-, B- AND C-level disease activity.  Index: BILAG | **CR:** 6/11 (54.5%)  **CR and PR**: 11/11 (100%)  **Median BILAG:** from 14 to 2 at 12 months (p<0.001)  **Relapses:** 7/11 (63.6%)  **Median duration of remission**: 12 months  **B-cell depletion**: 11/11 (100%) |
| **Tokunaga et al. 2007**  **Prospective cohort** | n = 10  Female: 10 (100%)  All patients had CNS involvement  Acute confusional state: n=5; Psychosis: n=4; Seizure: n=2; Headache: n=1; Mood disorder: n=2; Demyelinating syndrome: n=1; Cognitive dysfunction: n=1; Myelopathy: n=1; Anxiety disorder: n=1 | SLEDAI was determined before and after 1 – 6 months after treatment.  Clinical symptoms and treatment-induced adverse reactions were assessed before and every week during treatment.  Index: SLEDAI | **Improvement in neuropsychiatric manifestations:** 10/10 (100%)  **Complete recovery:** 4 (40.00%)  **Mean SLEDAI**: from 19.9 (range 2 - 49) to 6.2 (range 0 - 15)  **Relapses**: 6/10 (60%)  **Mean duration of remission**: 14 months (range 4 - 23)  **B-cell depletion**: 8/8 (100%) |
| **Jónsdóttir et al. 2008**  **Prospective cohort** | n = 16  Female: 16 (100%)  Mean age: 37 (range 19 – 56)  Nephritis: n=9; Arthritis: n=4; General: n=4; Serositis: n=3; Skin: n=3; Blood: n=3; Neurological: n=2; Vasculitis: n=2 | Global clinical response defined as a reduction of ≥50% in the SLEDAI score from baseline, or a reduction in the score of any organ system from BILAG A to B, or from B to C.  Remission was defined as a SLEDAI score <3, or as the absence of any BILAG A or B.  Relapse was defined as an occurrence of a new BILAG A or at least two new BILAG B in any organ system.  Index: BILAG and SLEDAI | **Mean SLEDAI (±SD) at 6 months:** 12.1 ± 2.2-4.7 ± 1.1 (p<0.050).  **BILAG at 6 months:**  BILAG A organ domains = 20, 100% progressed to ≤BILAG B.  BILAG B organ domains = 11. 8 (72.7%) organ domains progressed to ≤ BILAG C.  **Global clinical response (6 months):** 13/16 (81.3%). |
| **Lu et al. 2009**  **Prospective cohort** | n = 50  Female: 48 (96.0%)  Mean age: 32.8 years (range 15 – 73)  Arthritis: n=39; Skin: n=17; Serositis: n=16; Neuropsychiatric: n=6; Thrombocytopenia: n=4; AIHA: n=2; Antiphospholipid syndrome: n=2; Myositis: n=1; Gut vasculitis: n=1; Mouth ulcers: n=1 | Clinical outcomes were assessed every 1 – 3 months.  CR defined as a change from BILAG A or B score to BILAG C or D in every organ system.  PR defined as a change from BILAG A or B to a C or D score in at least 1 system, but with persistence of 1 BILAG A or B in another system.  NR defined as BILAG A or B that remains unchanged after treatment.  Index: BILAG | **Median BILAG at 6 months**: from 12 (IQR 8- 15.5) to 5 (IQR 2.5 - 7); p < 0.001  **CR at 6 months:** 19/45 (42.2%)  **PR at 6 months:** 21/45 (46.6%)  Median anti-dsDNA at 6 months: from 106 (IQR 21 - 455) to 42 (IQR 13.5 - 181) IU/ml; p = 0.0001  Median C3 at 6 months: from 0.81 (IQR 0.52 - 1) to 0.95 (IQR 0.8 - 1.3) g/l; p < 0.02 |
| **Catapano et al. 2010**  **Prospective cohort** | n = 31  Female: 28 (90.3%)  Mean age: 40.2 ± 12.8 years  Skin: n=30; Joint: n=30; Antiphospholipid syndrome: n=4; Ocular: n=12; Renal: n=13; Lung: n=13; CNS: n=13; Blood: n=5; Gut: n=2 | Response was assessed at 3, 6, 12 and 24 months after treatment.  CR defined as absence of BILAG A-, B-, and C-level disease activity.  PR defined as the absence of BILAG A- and B-level disease activity.  Relapse was defined as an increase in disease activity that required an increase in the prednisolone dose.  Index: BILAG | **CR after the 1st RTX course:** 27/31 (87%) at median time of 4 months (IQR 1-9).  **CR after the 2nd RTX course**: 15/16 (93.7%) at median time of 2 months (IQR 1-5).  **BILAG median change** (2-year follow-up) (n=18): from 14.5 to 3 at 12 months and 3.5 at 24 months (p < 0.001).  **Relapses:** 18/27 responders (67%) after a median of 11 months (IQR 4-24).  Anti-dsDNA: from 147 to 54 IU/ml at 24 months.  C3 and C4: before RTX C3 and C4 were low in 9 and 13 patients, respectively. C3 rose in 8/9 (88.8%) patients at 12 months from (mean ± SD) 0.52 ± 0.07 to 0.71 ± 0.09; p < 0.01) and in 7/9 (77.7%) patients at 24 months (from 0.52 ± 0.09 to 0.73 ± 0.1; p < 0.01).  C4 rose in 11/13 (84.6%) patients at 12 months (from mean (± SD) 0.08 ± 0.01 to 0.14 ± 0.01; p < 0.01) and in 7/13 (53.8%) patients at 24 months (from 0.08 ± 0.01 to 0.15 ± 0.01; p < 0.01). |
| **Terrier et al. 2010**  **Prospective cohort** | n = 136  Female: 111 (81.6%)  Mean (± SD) age: 39.1 ± 14.4 (range 9 - 87)  Skin: n=72; Joint: n=58; Kidney: n=42;  Blood: n=37; Serositis: n=18; CNS: n=10; Myocarditis: n=3; Peripheral neuropathy: n=3; Lung: n=2 | Response was assessed at 6 ± 3 months (mean ± SD) after the last RTX infusion.  Overall response defined as a reduction in the SELENA-SLEDAI score of ≥3.  Cutaneous response: ≥50% improvement (PR) or disappearance (CR) of baseline manifestations.  Articular response: ≥50% improvement in the number of painful and/or swollen joints (PR) or disappearance of pain and swelling from joints (CR).  AIHA: PR defined as a haemoglobin level >10 g/dl. CR defined as a haemoglobin level >11 g/dl in women and 12 g/dl in men without haemolysis.  Autoimmune thrombocytopenic purpura: CR defined as a platelet count of ≥100,000/mm3, PR defined as 30,000 – 100,000/mm3 and at least a doubling of the baseline count. NR defined as a platelet count <30,000/mm3 or less than doubling of the baseline count.  Index: SELENA-SLEDAI | **Mean (± SD)** **SLEDAI:** from 10.8 ± 8.8 to 3.4 ± 5.2 at 6 ± 3 months; p < 0.0001.  **Global clinical response**: 80/113 (71%).  No differences in patients treated with and without immunosuppressive agents concomitantly.  **Skin manifestations**: 48% CR and 23% PR  **Joint involvement**: 52% CR and 20% PR  **AIHA:** 69% CR and 15% PR.  **Idiopathic thrombocytopenic purpura**: 77% CR and 15% PR.  **Relapses**: 31/76 responders (41%) relapsed in 14.9 ± 7.6 months (mean ± SD).  **Mean (± SD) time-to-relapse**: 18.6 ± 13.5 months in patients with immunosuppressive agents vs. 13.5 ± 8.2 months in patients without concomitant immunosuppressive agents (p = 0.04). |
| **Vital et al. 2011**  **Prospective cohort** | n = 39  25/39 patients (64.1%) had BILAG grades of A in ≥1 domain  8/39 patients (20.5%) had BILAG grades of B in ≥2 domains.  5/39 patients (12.8%) had BILAG grade of B in only 1 domain but had disease that was resistant to alternative therapies and had more sever disease in the past. | Clinical response measured with the original BILAG index at baseline and every 3 months.  CR defined as no domain rated BILAG A or B at week 26 and no A or B flare between weeks 0 and 26.  PR defined as a maximum of 1 domain with a persistent B rating at 26 weeks with improvement in all other domains rated A or B at baseline, no new grade A flare between weeks 0 and 26, and no new grade B flare in more than 1 single domain between weeks 0 and 26.  NR defined as patients not meeting the criteria for CR or PR.  Relapse defined as a new BILAG grade A flare or 2 grade B flares following major clinical response or PR at 26 weeks.  Index: BILAG | **Week 26: CR 20/39 (51%), PR 12/39 (31%), NR 7/39 (18%).**  **Response in individual BILAG domains** (week 26):  **General:** CR 9/10 (90%), PR 1/19 (5%), NR 0%, A or B flares 0%.  **Mucocutaneous:** CR 12/19 (63%), PR 1/19 (5%), NR 6/19 (32%), A flares 1/19 (5%), B flares 2/19 (11%).  **Neurologic:** CR 12/13 (92%), PR 0%, NR 1/13 (8%), A flares 0%, B flares 1/13 (8%).  **Musculoskeletal**: CR 17/20 (85%), PR 2/20 (10%), NR 1/20 (5%), A flares 0%, B flares 3/20 (15%).  **Cardiorespiratory:** CR 7/7 (100%), PR 0%, NR 0%, A or B flares 0%.  **Vasculitis**: CR 6/6 (100%), PR 0%, NR 0%, A flares 0%, B flares 1/6 (17%).  **Haematologic:** CR 8/12 (67%), PR 0%, NR 4/12 (33%), A or B flares 0%.  **BILAG median change**: from 14 (IQR 9-23; n = 39) to 3 (IQR 2-5; n = 37) at 26 weeks and to 4 (IQR 2-8; n = 31) at 40 weeks (p < 0.0001).  **Relapses:** 24/28 (85.7%) patients with major or partial clinical response at 26 weeks and total follow-up of at least 18 months.  **Time-to-relapse:** earlier relapse at 12 months (n = 14) and later relapse at median 33 months (n = 10). |
| **Turner-Stokes et al. 2011**  **Prospective cohort** | n = 18  Female: 18 (100%)  Mean age: 29.9 years (range 17 – 57)  Joint: n=13; Kidney: n=12; Skin: n=10;  Serositis: n=6; AIHA: n=2; Alopecia: n=2; Mouth ulcers: n=1; Neuropsychiatric: n=1; CNS vasculitis: n=1 | Response was evaluated at 6 and 12 months following each cycle.  CR defined as a change from a BILAG A or B score to a C or D score in every organ system.  PR defined as a change from a BILAG A or B to a C or D score in at least one system, but with persistence of one BILAG A or B score in another system.  NR defined as a BILAG A or B score that remained unchanged after treatment.  Index: BILAG | **Median BILAG change after the 1st and 2nd cycle:**  6 months after the 1st cycle: from 12.5 to 8 (p < 0.01)  6 months after the 2nd cycle: from 13.5 to 8 (p < 0.01)  12 months after the 1st cycle: from 12.5 to 5 (p < 0.05)  12 months after the 2nd cycle: from 13.5 to 4 (p < 0.01)  **Time-to-relapse:** 38% within 6 months and 82% within 12 months after the 1st cycle. 45% within 12 months after the 2nd cycle. Time-to-flare was significantly prolonged following the 2nd cycle vs. the 1st cycle (X^2^ = 32.39; p < 0.01) |
| **Pinto et al. 2011**  **Prospective cohort** | n = 42  Female: 35 (83.3%)  Mean (±SD) age: 29.7 ± 8.9 years  Kidney: n=32; Neuropsychiatric: n=12; Blood: n=11; Musculoskeletal: n=10;  Cardiopulmonary: n=9 | Clinical and laboratory variables were measured before initiating therapy and every 3 months thereafter.  Response to treatment was evaluated through the reduction of the SELENA-SLEDAI score, as well as the number of subjects with hypocomplementemia (C3 and C4).  Neuropsychiatric SLE: response was evaluated through clinical neurologic exam.  Autoimmune cytopaenias: CR defined as haemoglobin levels of ≥12 g/dl and a platelet count of >150,000/mm3 without requiring additional corticosteroids within 3 months.  Index: SELENA-SLEDAI | **Significant reduction in SELENA-SLEDAI score**: reduction >60% at 3 months (p < 0.005).  Improvement in 9/12 (75%) neuropsychiatric symptoms and cytopaenias at 3 months.  The number of patients with hypocomplementemia (C3 and C4) decreased significantly after 3 months: from 26 to 5 patients and 25 to 5 patients, respectively). |
| **Fernández-Nebro et al. 2012**  **Retrospective cohort** | n = 128  Female: 115 (89.8%)  Mean (±SD) age: 38.2 ± 12.1 years  Musculoskeletal: n=42; Skin: n=25; Kidney: n=63; Blood: n=48; Neurological: n=27; Heart: n=18; Lung: n =22; Other: n=18 | Rate of either CR or PR at 6 ± 3 months after the first course of RTX.  CR defined as a SELENA-SLEDAI score of ≤2 points and a modified SELENA-SLEDAI Flare Index score of 0.  PR defined by a reduction of at least 4 points in the SELENA-SLEDAI score with no new or worsening symptoms as measured by the SELENA-SLEDAI Flare Index.  Index: SELENA-SLEDAI | **Response rate at 6 ± 3 months after the 1st course of RTX:**  CR or PR: 73/116 (62.3%; 95% CI 49.3-79.1)  CR: 22/116 (19.6%; 95% CI 12.3-29.7)  PR: 51/116 (45.5%; 95% CI 36.1-55.2)  **Clinical and serological response at 6 ± 3 months after 1st course of RTX**:  Mean (±SD) SELENA-SLEDAI (n = 116): from 14.6 ± 10 to 4.8 ± 4.5, p < 0.001.  **Joint involvement** (n = 45): from 54 (42.2%) to 4 (3.5%) patients, p < 0.001  **Skin manifestations** (n = 31): from 31 (24.2%) to 8 (7%) patients, p < 0.001  **Nasopharyngeal ulcers** (n =10): from 10 (7.8%) to 3 (2.6%)( patients, p = 0.109  **Thrombocytopaenia** (n = 21): from 21 (16.4%) to 3 (2.6%), p < 0.001  **Haemolytic anaemia** (n = 6): from 6 (4.7%) to 1 (1%), p = 0.125  **Systemic vasculitis** (n = 6): from 6 (4.7%) to 2 (1.7%), p = 0.063  **Pleuritis** (n = 8): from 8 (6.3%) to 1 (1%), p = 0.031  **Pericarditis** (n = 7): from 7 (5.5%) to 2 (1.7%), p = 0.031  **Fever** (n = 17): from 17 (13.3%) to 3 (2.6%), p = 0.001  **Response rate at the end of follow-up**:  CR or PR: 97/125 (77.6%; 95% CI 62.9-94.7)  PR: 47/125 (38.5%; 95% CI 29.8-47.8)  **Median time to achieve best response** (n = 125): 6.5 months (IQR 5-8)  **Relapse rate following the 1st course of RTX**: 37 patients (38.1%; 95% CI: 26.8-52.6) after a median of 10.8 months.  **CR or PR at the end of follow-up:**  Arthritis: 93%  Skin: 87.5%  Neuropsychiatric 73%  Thrombocytopenia: 65%  Severe generalised flare: 62% |
| **Witt et al. 2013**  **Retrospective cohort**  **(GRAID registry)** | n = 85  Female: 69 (81.0%)  Mean age: 36.6 years  Fatigue: n=41; Erythema: n=36; Anemia: n=33; Myalgia: n=29; Arthritis: n=25; Kidney: n=31 | Efficacy assessments were restricted to a categorization of CR, PR and NR as judged at the discretion of the treating physician.  Response was further evaluated by comparison of mean SELENA-SLEDAI scores at baseline and after last infusion.  Index. SELENA-SLEDAI | **CR**: n = 37 (46.8%)  **PR**: n = 27 (34.2%)  **NR:** n = 15 (19.0%)  **Mean SELENA-SLEDAI** scores decreased significantly from 12.2 to 3.3 during rituximab treatment (p<0.05).  **Presence of signs and symptoms**: Improvement in major manifestations (p < 0.05 each). Fever: 20.0% to 8.2%. Weight loss: 11.8% to 2.4%. Fatigue: 48.2% to 28.2%. Skin symptoms: 42.4% to 21.2%. Mucocutaneous involvement: 21.2% to 10.6%. Raynaud’s syndrome: 35.3% to 18.8%. Pleuritic symptoms: 12.9% to 3.5%. Anemia: 38.3% to 23.5%. Leucopenia: 20.5% to 9.1%. Thrombocytopenia: 32.9% to 11.8%. Glomerulonephritis: 36.5% to 21.5%. Other manifestations such as musculoskeletal and neurologic manifestations also improved but did not reach statistical significance.  The proportion of patients with leukopenia was reduced significantly from 20.5% to 9.1%  (p<0.05).  Mean complement C3 and C4 levels increased from 68.3 to 77.5 mg/dl and from 10.8 to 12.4 mg/dl, respectively (not significant).  The proportion of patients with elevated dsDNA antibody levels decreased from 73.4% to 62.2% (not significant). |
| **Gómez et al. 2018**  **Retrospective cohort** | n = 20  Female: 16 (80.0%)  Mean (±SD) age: 43.9 ± 15.8 years  Arthritis: n=10; Kidney: n=8; Hematologic: n=6; Cutaneous: n=5; Pericarditis: n=3; Neurologic: n=1 | Results were evaluated before and 3 months after each cycle of RTX.  Clinical and laboratory variables were collected, as well as SLEDAI before and after treatment.  Index: SLEDAI | **Median SLEDAI** Before: 10 (IQR 8-12); After: 4 (IQR 2-8) (p<0.001).  Median ESR Before: 33.5 (IQR 15.2-54.7); After: 18.5 (IQR 5.7 – 27.7) (p = 0.017)  Significant improvement of C4 levels after treatment (p = 0.014)  No significant reduction of anti-dsDNA (p = 0.125) |
| **Cassia et al. 2019**  **Retrospective cohort** | n = 147  Female: 134 (91.2%)  Mean age: 44 (range 41 – 46)  Musculoskeletal: n=87; Skin: n=80; Hematologic: n=63; Lung: n=24; Kidney: n=52; Neurologic: n = 22 | Results were assessed 6 months after the first RTX course.  CR was defined as a physician assessment of disease activity score of 0 or 1, a reduction in the ECLAM score of ≥50%, and a decrease in the dose of immunomodulating agents of ≥25% from baseline.  PR was defined as a physician assessment of disease activity score of >0, a reduction in the ECLAM score of 25 – 50%, and a decrease in the dose of immunomodulating agents of 0 – 25%.  Any other response was defined as a NR.  Disease flares were defined as an increase in at least 2 of the 3 parameters (physician assessment of disease activity, ECLAM score, and number or dose of immunosuppressive agents or glucocorticoids). | **CR:** 67 patients (45%)  **PR:** 41 patients (28%)  **NR:** 39 patients (27%)  **Mean ECLAM score**: from 4 (95% CI 3.65 – 4.34) to 1.9 (95% CI 1.66 – 2.14) at 6 months; p < 0.0001.  Of the 67 patients who received a single course of RTX, 54 (80%) achieved a CR or PR, and 18 (33%) experienced a disease flare (51.5 flares per 100 patient-years).  Of the 80 patients that received RTX as a maintenance treatment, 28 (35%) did not experience any flares during the maintenance period and were classified as sustained responders. Follow-up data were available for 21 of the 28 sustained responders. Ten (48%) of the 21 patients experienced a flare (27 flares per 100 patient-years). |

*Patients could have more than one clinical manifestation of SLE.

AIHA: autoimmune haemolytic anaemia; anti-dsDNA: anti-double stranded DNA antibodies; BILAG: British Isles Lupus Assessment Group; CNS: central nervous system; CR: complete response; C3: complement component 3; C4: complement component 4; ECLAM: European Consensus Lupus Activity Measurement; ESR: erythrocyte sedimentation rate; IQR: interquartile range; NR: no response; PR: partial response; RTX: rituximab; SD: standard deviation; SLE: systemic lupus erythematosus; SELENA-SLEDAI: Safety of Estrogens in Lupus Erythematosus National Assessment- Systemic Lupus Erythematosus Disease Activity Index; SLEDAI: Systemic Lupus Erythematosus Disease Activity Index.
